# Supplementary material for: Analysis of growing season drought characteristics and driving factors for vegetation in the Santun River Irrigation Area in Xinjiang
Source: PLoS One. 2025 May 13;20(5):e0323918. doi: 10.1371/journal.pone.0323918 (PMC12074591; doi:10.1371/journal.pone.0323918)
Supplement: Attachment — Submitted filename: Response to Reviewers.docx [file pone.0323918.s004.docx]

**Response to Reviewers**

Thank you for your letter. We would like to thank the reviewers and editorial department for their comments on our manuscript. We have revised the manuscript with reference to the reviewer's comments for your approval. Our replies to the reviewers’ comments are presented below.

**Reviewer #1**

1.The introduction of the manuscript emphasizes the insufficiency of current research in integrating human activities with drought issues. However, the discussion on the impacts of land use changes and human activities on drought remains superficial, lacking an in-depth exploration of the underlying driving mechanisms. Furthermore, the study fails to adequately address the core questions raised in the introduction.

Answer: Based on your suggestions, we have reorganized the discussion section of the paper to focus on in-depth discussion of the impacts of human activities and land use change types on drought and to explore in depth the underlying driving mechanisms. **Please refer to lines 474–478 and 489-512.**

Regarding the issue of the introduction, after an in-depth discussion between members of the team, we have reorganized the introduction and core issues of this paper. The core of this paper was calculating the temperature vegetation drought index (TVDI) in the study area and to clarify the spatial and temporal distribution characteristics of drought and the influencing factors of drought in the Santun River Irrigation Area in Xinjiang during 2005**–**2023 using a combination of trend analysis, Geodetector, and spatial transfer matrix so as to provide a scientific basis for optimizing the allocation of water resources, adjusting the irrigation system, and guaranteeing the stability of agricultural production. Therefore, we have revised the introductory section of this paper to ensure logical consistency between the research objectives and scope of the study. **Please refer to lines 45-51,58-61 and 66–95.**

2.While the paper employs the TVDI indicator, which is suitable for areas with substantial vegetation cover, its applicability in regions with sparse vegetation or bare soil is debatable. The manuscript does not sufficiently address the limitations of this indicator or its potential impact on the results. A more thorough discussion of these limitations would enhance the credibility of the findings.

Answer: Based on your suggestions, we have reorganized the discussion section to focus on adding a discussion of the potential impacts of the limitations of the TVDI metrics on the results. **Please refer to lines 581–598.**

3.The study primarily relies on annual temporal resolution. Although seasonal variations are mentioned, the analysis does not capture critical short-term drought fluctuations within key growth periods, which diminishes its direct applicability to agricultural production and water resource management. Additionally, the use of a 30-meter spatial resolution, though appropriate for large-scale regional analysis, may obscure local heterogeneity in small-scale irrigation areas, reducing its utility for precise monitoring and strategy development.

Answer: Thank you for your valuable comments. We agree with you. We also recognize the shortcomings in the research and sincerely thank you for your correction. However, since current research on drought characteristics and influencing factors in the Santun River Irrigation Area in Xinjiang is still lacking, the main purpose of this paper is to fill research gaps in the study of drought in this region, to provide basic data and preliminary understanding for subsequent related research, and to pave the way for later refinement of research on the irrigation district. Therefore, we chose Landsat series data, which are widely applicable, have a long time series, are easy to obtain, and have comprehensive information on spectral bands, to analyze the long time-series drought characteristics and influencing factors in the Santun River irrigation area in Xinjiang. In response to the issues you mentioned, we plan to further improve the temporal and spatial resolutions of the data (Sentinel-2 and ground-based observations) in future studies, and to refine the analysis of droughts in the region by considering factors such as the type of cultivation in the irrigation area and the irrigation system in order to capture critical short-term drought fluctuations during the crop growth period and to improve the sensitivity and ability to identify localized heterogeneity in small-scale irrigation areas. Following your suggestion, we have added a discussion of this deficiency in Section 4. **Please refer to lines 06–611.**

4.Although the manuscript employs the GeoDetector model to reveal the independent and interactive effects of various driving factors, the explanations of these interactions remain qualitative, lacking robust scientific reasoning. Specifically, the paper identifies the interaction between elevation and temperature as having the strongest explanatory power for drought distribution but fails to elucidate the underlying physical mechanisms behind this phenomenon.

Answer: Following your suggestion, we have reorganized and deepened the discussion of the drivers in this article and have added a discussion of the underlying physical mechanisms behind this phenomenon. **Please refer to lines 541–556.**

5.The introduction highlights the large-scale impacts of drought and critiques the limitations of large-scale studies in integrating human activities and regional heterogeneity. However, the research itself is focused on the Santun River Irrigation Area, a small-scale region, analyzing local drought characteristics and drivers. This shift between the research objectives and study scope lacks clear logical coherence, resulting in a degree of inconsistency in the manuscript.

Answer: Thank you for your valuable comments. We have revised the introduction section of this paper to ensure logical consistency between the research objectives and scope of the study. **Please refer to lines 45-51,58-61 and 66–95.**

6.The correlation analysis between TVDI and soil moisture does not report p-values to verify the strength of the relationship. The GeoDetector model results, including single-factor (e.g., temperature, NDVI) and interaction effects, lack significance tests to confirm the robustness of the q-values. The Mann-Kendall trend analysis for seasonal and annual TVDI variations omits the significance levels (e.g., p-values) of the identified trends.

Answer: According to your suggestion, we have added the p-value to the correlation analysis between the TVDI and soil moisture in the text. **Please refer to lines 211–212**. In the single-factor analysis of the geographic detector, the p-value was added, and the original q-value map was replaced with a single-factor analysis table. **Please refer to lines 396 and 407**. We reorganized the data and added the significance level in the trend analysis. **Please refer to lines 339–345.**

7.In the discussion of seasonal drought variations, the reasons for the intensification of spring drought and the alleviation of summer drought are not analyzed in depth. The explanation is limited to temperature changes or irrigation water use, without considering factors such as precipitation distribution or adjustments in crop planting structures. Additionally, for the spatial heterogeneity where drought is more severe in the northern region, it is merely described as being "close to the desert with sparse vegetation," without a detailed analysis of human activities (e.g., over-extraction of water resources, urban expansion) or natural factors (e.g., soil water retention capacity).

Answer: Based on your suggestion, we have reorganized the discussion section to deepen the discussion on the causes of the seasonal drought. In addition, the discussion of the arid spatial heterogeneity phenomenon has been added to the discussion of human activities, natural factors, and other content. We have made the content more substantial and persuasive. **Please refer to lines 474–478 and 489-512.**

**Reviewer #2**

1. What is the difference between the canopy temperature in VSWI and LST?

Answer: Thank you for your valuable comments. The main parameter used in this study is the TVDI, and the one used is based on the surface temperature (LST) obtained by thermal infrared sensors carried by the Landsat series of satellites.

The VSWI is calculated using the canopy temperature, which is the temperature of the plants and/or vegetation at the surface and is commonly used to indicate the heat and moisture status of surface vegetation. Meanwhile, the TVDI is calculated using the land surface temperature (LST), which is an important parameter for heat exchange at the interface between the atmosphere and the land surface and reflects the degrees of heating and cooling of surface objects. The main difference between the two are discussed below.

1) The measurement object is different: the canopy temperature mainly measures the temperature of the crop canopy (such as stems and leaf surfaces), which more directly reflects the moisture and heat status of the crop itself. The surface temperature includes the temperature of the entire surface, including soil, vegetation, and water bodies. In remote sensing applications, it is usually measured as the temperature of the entire surface, including the soil, vegetation, and water bodies. In remote sensing applications, it usually represents the average or composite temperature over a wider area.
2) Different influencing factors: The canopy temperature is influenced by a variety of factors, including the soil moisture status, crop transpiration, and environmental factors (e.g., wind speed, temperature, and solar radiation). The surface is more strongly influenced by the solar radiation, type of ground cover, atmospheric conditions, and other factors.
2. Are there any other similar studies examining the small scales other than the study area mentioned in the paper?

Answer: Based on a comprehensive review of the relevant literature, I observed that relevant studies conducted on small scales such as irrigated areas exist. The specific relevant studies are listed below.

1) Yue Qiong et al. utilized the CFSMP model to analyze the seasonal drought in irrigation areas in South China. Their results showed that the CSFMP model has the potential to mitigate seasonal drought in South China and can be applied to similar regions with comparable resource crises (Reference: Qiong Y, Ningbo C, Fan Z, et al. Adaptation to seasonal drought in irrigation districts of south China: A copula-based fuzzy-flexible stochastic multi-objective approach for precise irrigation planning [J]. Journal of Hydrology, 2023, 625 (PA):).
2) Zhang Fan et al. proposed a Copula-based stochastic multi-objective planning (C-SMP) model for optimizing irrigation strategies to mitigate the negative impacts of seasonal agricultural drought on the yields of different crops in the irrigation area of the Dongfeng Reservoir, Meishan City, Southwest China (Reference: Fan Z, Ningbo C, Shanshan G, et al. Irrigation strategy optimization in irrigation districts with seasonal agricultural drought in southwest China: A copula-based stochastic multiobjective approach [J]. Agricultural Water Management, 2023, 282).
3) Akinwale T. et al. conducted spatiotemporal characterization of drought in northern Nigeria using the self-calibrated Palmer drought severity index (sc-PDSI) (Reference: Ogunrinde T A, Oguntunde G P, Olasehinde A D, et al. Drought spatiotemporal characterization using self-calibrating Palmer Drought Severity Index in the northern region of Nigeria [J]. Results in Engineering, 2020, 5 (C): 100088-100088.).

In summary, research has been conducted on drought characteristics at small scales or within specific regions. Our study clarifies the characteristics and influencing factors of drought in the Santun River Irrigation District in Xinjiang using trend analysis, Geodetector, and spatial transfer matrices. This is crucial for optimizing water resource allocation in the irrigation area, ensuring stable agricultural production, and promoting sustainable agricultural development.

3. There are a number of abbreviations in the text, and it is recommended that a table or an appendix be added listing all abbreviations and their full names.

Answer: Thank you for your comments. The full names and abbreviations have been added in the form of a table. **Please refer to line 128.**

4. Line 130: amax and bmax should use suffix notation.

Answer: This has been modified. **Please refer to line 148.**

5. Line 131: amin and bmin should use suffix notation.

Answer: This has been modified. **Please refer to line 148.**

6. Line 281: 2020 should be 2023

Answer: This has been modified. **Please refer to line 322.**

7. Reference 34 and 42 are the same

Answer: Thank you for your comments. This has been modified.

**Reviewer #3:**

1. Data sources are not clearly specified. There is a lack of descriptions regarding the time range and spatial resolution of the data sources in Table 2. The authors mention choosing 60 images from 2005 to 2023, but the frequency of the dataset seems low. The authors need to justify whether these calculation results can represent quarterly drought characteristics.

Answer: Thank you for your comments. In response to your suggestion, we have modified Table 1 to add the time ranges of the data. **Please refer to line 126.** The selection of the dataset was based on strict selection criteria after excluding effects such as cloudiness and weather, and we ensured that the selected images were evenly distributed in time and covered key periods in each season to ensure that they accurately reflect the drought conditions in each season. In addition, we compared the results of the remote sensing calculations with the soil water content observed on the ground and found that there was a good correlation between the two, indicating that our remote sensing calculations can better reflect the drought conditions on the ground. Compared with similar studies, we believe that the datasets used in this study are more accurate.

2. Similarly, the soil moisture data in Table 2 need to include information about its spatial extent. If these data are discrete, the interpolation methods also need to be described.

Answer: According to your recommendations. The measured soil moisture content data used in this study are continuous data. We have added information on the spatial extent of the sampling points for the measured soil moisture content in the irrigation area (Figure 3). **Please refer to lines 203–205.**

3. The paper focuses on vegetation area, but some areas without vegetation are also included in the statistics, such as urban-rural residential land, water bodies, and unused land. Excluding these areas might yield more accurate results.

Answer: Thank you for your valuable comments. During the analysis conducted in this study, we mainly considered that non-vegetated areas are equally important for studying the functioning of the overall ecosystem and the effects of drought as they may indirectly affect the growth and distribution of vegetation. For example, urban and rural residential land use may indirectly affect the surrounding vegetation by changing the surface cover and affecting the hydrological cycle. Therefore, we have discussed and inferred about this in the discussion section. **Please refer to lines 489–512.** In a follow-up study, we will use more precise geographic information technology tools to identify and extract vegetation areas to ensure the accuracy of the study. Thank you again for your review and guidance.

4. In section 3.3.1, the paper statistically analyzes the annual average TVDI and the percentages of the area with different drought classes from 2005 to 2023. The methodology and its rationale should be explained; additionally, the paper should clarify how the calculated drought areas were validated.

Answer: Thank you for your comments. In this study, the method used to analyze the change trend of the annual average TVDI was linear trend analysis. The analysis of the percentages of the different drought types was mainly conducted using ArcGIS to extract the data in the raster image followed by statistical analysis in Excel. After integrating the data, we plotted the percentage of the stacked area using the Origin software. Based on your suggestion, we have added instructions on how to use it in our analysis. **Please refer to lines 248–250.** The validation of the credibility of the results of this study was carried out using the measured soil water content and drought indicators for credibility evaluation, combined with analysis of the results of the dry and wet edge fitting. In addition, we verified the calculation results through visual comparison against the *Chinese Disaster Dictionary - Xinjiang Volume*.

5. The explanation for Fig 11 is unclear, with no introduction of the numbers before the area percentages. Additionally, Fig 11 (a) and Fig 11 (b) are both labeled "Irrigation area transfers," while Fig 11 (c) and Fig 11 (d) are labeled "Irrigation district drought transfers." The descriptions should be consistent.

Answer: Based on your recommendations, we have reorganized the expression and have introduced a number before the percentage of the area. **Please refer to line 371.** We have harmonized the names in Figures 12(a) and 12(b) to read “irrigation district drought transfers.” **Please refer to lines 387–390.**

6. Section 3.4 analyzes the influence of driving factors but fails to explain their rationale and calculation methods. Moreover, precipitation, discussed as an influential factor in sections 4.1 and 4.2, is not mentioned among the driving factors.

Answer: We apologize for any misunderstanding caused by our unclear wording. The basic principles and methods of the driver analysis have been listed in Subsection 2.3.3. **Please refer to lines 175–195.** Regarding the issue that precipitation was mentioned in the discussion section but not in the driver analysis section, this is mainly due to the following reasons.

1) In writing the paper, we tried to focus on the drivers that have the most direct and significant impact on the results of the study. The Xinjiang Santun River Irrigation District is located in the hinterland of the Asia-Europe continent, which is a typical high-altitude arid and semi-arid region. The main water for production and domestic use is glacier meltwater and groundwater, and the precipitation does not have a very high degree of influence in this region.

2) The study area is small compared to larger scale areas. There is only one meteorological station in the study area, so the precipitation data cannot be used to represent the entire study area as this would reduce the accuracy of the analysis.

3) In the driver analysis section, the method used was Geodetector-driven analysis. The required data for this method are raster data. It is very unfortunate that during the data research phase of this study, after careful investigation, we found that due to the special geographic location and size of the study area, in the existing remote sensing raster data, the precipitation data for the area have very small variations; and by comparing these data with the meteorological station data we found that there is a large gap between the two, so these data cannot accurately represent the regional precipitation situation.

In summary, we did not consider precipitation when writing the driver analysis section of the paper. However, there is also a relationship between the occurrence of drought hazards and precipitation factors in the study area, so in order to analyze and discuss this issue more comprehensively, we have also mentioned this factor appropriately in the discussion section in Section 4. We appreciate your suggestions and will continue to work on them in our subsequent studies. Thank you again for your review and guidance.

7. Some discussions in this manuscript are inadequate. The inference of drought driving factors in section 4.3 and the discussion of drought response strategies in section 4.4 should focus on the drought driving factors studied in this paper. The current arguments seem disconnected from the research content and should be revised to align with the research findings.

Answer: According on your recommendations. We have reframed the discussion in Section 4.3 to focus primarily on the drought drivers in this study and to scientifically investigate the underlying physical mechanisms. **Please refer to lines 541–556.** In response to the discussion of drought response strategies in Section 4.4, we focus on how to develop effective drought prevention and mitigation measures based on the key drivers identified in this paper. In addition, we added a part of the practice-oriented content to propose some specific and feasible drought response strategies. **Please refer to lines 564–576.**

8. Some reference formats are inconsistent with the journal’s requirements and should be checked and revised.

Answer: Thank you for your comments. This has been modified.
