# Supplementary material for: Analysis of growing season drought characteristics and driving factors for vegetation in the Santun River Irrigation Area in Xinjiang
Source: PLoS One. 2025 May 13;20(5):e0323918. doi: 10.1371/journal.pone.0323918 (PMC12074591; doi:10.1371/journal.pone.0323918)
Supplement: Attachment — Submitted filename: Review Comments.docx [file pone.0323918.s003.docx]

**Reviewer Comments.**

Overall, this research piece of work is very relevant in the context and time of climate change because it primarily impacted humans negatively. Still, there is a requirement for some change to make this piece of work more academically sound for the reader.

- In the abstract, the significance and conclusion sections are given less emphasis, typically appearing at the end. Meanwhile, the results and methodology sections are overly detailed. To balance the abstract, some content from these sections should be condensed and incorporated into the significance and conclusion.
- In the Introduction, the discussion lacks literature on the driving forces of drought and its impact on agriculture and human development. Instead, it focuses only on transitioning to new methods/models. Given the title's emphasis on driving forces, incorporating this aspect would enhance clarity and provide a better understanding for readers.
- A strong rationale is needed for using NDVI and TVDI in this study. Why are these indices essential, and how does this study contribute to your research area? While TVDI is widely used, its limitations in sparsely vegetated or bare soil regions should be explicitly acknowledged. Additionally, comparing it with other drought indices (SPI, SPEI, VHI) would enhance clarity.
- The interaction between elevation and temperature (q-value = 0.869) is identified as the strongest factor, but the underlying physical mechanisms are not well explained also the Geo Detector results lack significance testing (p-values, confidence intervals), which should be included for better validation.
- Why did you select only the 2005–2023 period? Provide a clear justification for this timeframe, explaining its relevance to the study. Clarifying this choice will also strengthen the trend analysis.
- The study links severe drought in the northern region to natural factors but overlooks human-induced impacts like groundwater extraction and irrigation inefficiencies. It should also discuss local drought mitigation efforts. Additionally, the conclusion needs revision to restate the topic and provide concrete recommendations

**Recommendation**

- **Minor Revisions Required**: The study is valuable but would benefit from clearer justification of TVDI, stronger discussion of drought-driving mechanisms, and better statistical validation of GeoDetector results. Addressing these points will significantly enhance the manuscript’s clarity and impact.
