# Supplementary material for: Analysis of growing season drought characteristics and driving factors for vegetation in the Santun River Irrigation Area in Xinjiang
Source: PLoS One. 2025 May 13;20(5):e0323918. doi: 10.1371/journal.pone.0323918 (PMC12074591; doi:10.1371/journal.pone.0323918)
Supplement: Attachment — Submitted filename: Response_to_Reviewers_auresp_2.docx [file pone.0323918.s005.docx]

**Response to Reviewers**

Thank you for your letter. We would like to thank the reviewers and editorial department for their comments on our manuscript. We have revised the manuscript based on the reviewer's comments and are submitting it again for your approval. Our replies to the reviewers’ comments are presented below. (Note: The line numbers in “Please refer to lines .” are from the Manuscript file.)

**Reviewer #4**

The study titled “Analysis of Growing Season Drought Characteristics and Driving Factors for Vegetation in the Santun River Irrigation Area in Xinjiang” presents a remote sensing-based assessment of drought trends. The manuscript very well articulated the drought analysis in arid areas of Xinjiang. It also addressed the research applicability and drought management strategies. However, there are certain suggestions that the author should take into account:

1. In the introduction section, the manuscript mentioned the study of droughts and its approaches but lacks a clear research gap and objectives that the study aims to address.

Answer: Based on your suggestions, we have rewritten the introduction of the paper to enhance the description of the shortcomings of drought research in the present stage and to clearly present the research objectives of this study. In addition, we have appropriately added research on drought-driven mechanisms, making the introduction more closely related to the topic of this paper. **Please refer to lines 76-109.**

2. The introduction deals more about the methods, such as TVDI, Theil-Sen trend analysis, etc., which should be appropriate for the methods section. The introduction should focus on the “how” aspect rather than “what” and "why," conveying the novelty of the research.

Answer: After in-depth discussion by the team, we have optimized the introduction section of the paper. We have streamlined the detailed description of the specific indicators and methods, focusing instead on how drought hazards should be studied and the current research progress. **Please refer to lines 34-64.** In addition, we have supplemented the discussion of the driving mechanisms of drought to ensure that the introduction is closely related to the topic of the article. **Please refer to lines 65-75.** On this basis, we now clear point out the shortcomings of the existing research and the shortcomings of the research area so as to naturally lead to the core goal of this study. Through these adjustments, we aimed to better highlight the innovation and value of this research.**Please refer to lines 76-109.**

3. Mention briefly why Santun RIA is significant for studying drought characteristics and its driving factors.

Answer: I have thought deeply about the questions you have posed and respond to them in detail here. The Santun River Irrigation District in Xinjiang is important for the study of drought characteristics and its drivers. The main reasons for this are presented below.

First, the Santun River Irrigation District in Xinjiang is located in the arid zone on the northern slopes of the Tianshan Mountains where water resources are scarce and the ecosystems are fragile, and the impact of drought on agricultural production and the ecological environment is significant. In addition, the discussion section of this paper also points out that the land use types in this area have changed significantly in the last 20 years, and this nonlinear interaction between natural and human factors provides an ideal case for analyzing the drought-driving mechanism.

Second, as the core area of the agricultural economy in Changji City, Xinjiang, the Santun River Irrigation District in Xinjiang is mainly dominated by high water-consuming crops, such as cotton and wheat, and studying the spatial and temporal differentiation patterns of drought in this region can reveal the response threshold of oasis agricultural systems to climate change and provide a scientific basis for optimizing the irrigation system and planting structure.

Finally, most existing studies focused on large-scale regions (e.g., the North China Plain and the Tibetan Plateau in China), and there is a relative lack of detailed studies on the north slope of the Tianshan Mountains, which is a key area of ecological-economic synergy. The study of the Santun River Irrigation District can fill the regional gap in drought-driving research and provide a reference for sustainable development in similar arid zones.

In summary, the Santun River Irrigation Area in Xinjiang is of great significance for studying drought characteristics and its driving factors, and it is a very good representative area. To prevent readers from having doubts, based on your suggestions, we have pointed out the importance of the research area and the existing research gaps, as well as the research objectives of this study, in the introduction of the paper. **Please refer to lines 87-91.**

4. In the methodology section, briefly mention the rationale behind choosing the mentioned methods for easy reproducibility of the work.

Answer: Thank you for your review. A rationale for the selection of each method has been added to the research methods section of this paper. **Please refer to lines 171-175, 189-193, 212-215, 221-225.**

5. The methodology section outlines various methods but does not justify why these were chosen for drought analysis.

Answer: Based on your suggestion, the reasons for choosing each method have been added to the research methods section of this article. **Please refer to lines 171-175, 189-193, 212-215, 221-225.**

6. The study analyzes the drought conditions over a specific period, but it could be improved by exploring the implications for future water resource management and agricultural planning.

Answer: We have reworked the section on coping strategies in the discussion section of the paper based on your suggestions. **Please refer to lines 565-582.**

7. The paper did not analyze the groundwater depletion in detail, which has been discussed as key factors for drought intensification in Xinjiang. Include the suggestion in the future research section.

Answer: Thank you for your advice. The importance of groundwater depletion factors has been added to subsection 4.4 of the discussion section. **Please refer to lines 604-606.**

8. The manuscript does not address human-induced factors such as urban expansion, irrigation, and policies.

Answer: Based on your suggestions, we have revised the discussion section to deepen the discussion of the influences of human factors on drought. In addition, in subsection 4.1 of the discussion section, we discuss the effects of human activities (e.g., changes in land use types and urban expansion) on drought. Appropriate reference is now made to the effects of drought on irrigation systems and groundwater extraction.**Please refer to lines 518-536; .**

In addition, in the analysis of the driving factors in the results section, we also considered human activities (land use type change and gross domestic product) and included them in the analysis of the driving factors. However, the effect of human activities is not as great as the influence of the natural factors on the drought. **Please refer to lines 447-480.**

**Reviewer #5**

I see articles have been revised well as per comments given in previous review. The topic undertaken is needs of the time and methods applied are relevant. The article is nicely designed and written well but I suggest authors to do a comprehensive language check as at several places English language is poor and sentences are not clear. Also, discussion needs a comprehensive revision as it is lengthy and did not provide a meaningful discussion. My specific comments are as:

1. Kindly avoid using personal pronouns like ‘we’, ‘you’, ‘our’, ‘I’, ‘us’, etc.

Answer: Thank you for the suggestions, we have revised the paper as a whole with reference to the comments of the reviewers and have enhanced the logic and accuracy of the language of the article. We have also hired a professional language editing service to thoroughly check and touch up the manuscript.

2. Second sentence in abstract may be revised.

Answer: We have revised the abstract of the paper in strict accordance with the "background, purpose, methods, results, and conclusion" framework for the summary part of the writing. The description of the methods in the second sentence of the abstract has been simplified to balance the overall structure of the abstract. **Please refer to lines 14-30.**

3. Keywords must be revised. The keyword ‘remote sensing monitoring’ did not make a clear meaning while the full form of TDVI should be written in keyword. Similarly, drought and Landsat are also not reliable as keywords, instead it may be written as ‘drought monitoring’ and ‘Landsat datasets’.

Answer: This has been modified accordingly.**Please refer to lines 31-32.**

4. Line 73. Add citation.

Answer: We have reorganized and rewritten the introduction section of the article. Where relevant citations needed to be added, we have rechecked and added them. **Please refer to lines 34-109.**

5. Table 1. Kindly add a column for each Landsat satellite data used.

Answer: As there are too many remote sensing images involved in this paper, this information cannot be clearly expressed in Table 1. After discussion among the team, we decided to organize each data image information used into a remote sensing image information table, which is convenient for readers to read and understand. The detailed information is presented in Table 2. In addition, sensor models designed in the image are also noted in Table 2. **Please refer to lines 154-157.**

6. Table 2 may be removed and the full form of each abbreviation used should be mentioned in manuscript at first appearance.

Answer: According to your suggestions, we have removed this table and define each acronym the first time it is used in the article.

7. Quality of figure 3, 9 and 10 should be improved.

Answer: Based on your suggestions, we have redrawn Figures 3, 9, and 10 in the paper. We added additional boundary conditions and improved the quality of the images. **Please refer to lines 268, 374, 377.**

8. Some studies have been done to monitor drought using Landsat data and temperature and vegetation indices. Authors should check these studies and compare the findings with these studies in the manuscript. Some of the studies are:

https://doi.org/10.1016/j.ejrh.2024.101689, https://doi.org/10.1007/s10661-022-10028-5, https://doi.org/10.1016/j.ecolind.2023.110584, https://doi.org/10.1007/978-981-19-3567-1_4, https://doi.org/10.1016/j.ecolind.2024.112681

Discussion seems to be a summary especially sub-section 4.2-4.5. Authors should try to build arguments based on the findings of this study and comparing it with previous studies. Kindly try to provide causes and effects of major findings and add a sub-section of policy implication at the end. In current form the discussion is lengthy but did not provide a concrete discussion.

Answer: Regarding the several research papers you mentioned, I have carefully read them and have referenced them and similar studies in the discussion section of this paper to support the research results and discussion. **Please refer to lines 491, 496, 505, 509, 519, 525, 535, 536, 542, 549, 558, 561.**

After discussion by the team, we decided to carry out block discussion according to the paper’s framework. This may make it easier for the reader to read and understand. In addition, we have made specific arguments based on the findings and similar studies in each section of the discussion. The discussion on the causes and effects of the main research findings is scattered in each section and mainly analyzes the reasons and driving mechanisms behind this phenomenon. **Please refer to lines 488-582.**

In addition, based on your suggestions, we provide policy recommendations and recommend drought response strategies in Section 4.3. to make it easier for readers to read and understand. **Please refer to lines 564-582.**

We have fine-tuned the discussion by reducing unnecessary narrative and refining the language. We hired a professional language editing service to thoroughly check and polish the manuscript.

9. Kindly bring whole conclusion in one or two paragraph.

Answer: Thank you for your suggestion. After discussion by the team, we decided to summarize the research according to three points, which may make it more convenient for readers to read and understand. In addition, according to your suggestion, we have simplified the conclusion and summarized the main conclusions it in concise language. In addition, in the last paragraph, we have also added appropriate statements on policy recommendations in accordance with other reviewers' comments to provide an overall conclusion to the article.**Please refer to lines 620-643.**

**Reviewer #6**

I have read the draft “Analysis of growing season drought characteristics and driving factors for vegetation in the Santun River Irrigation Area in Xin jiang” revised based on the comments received earlier from three reviewers. I have found that the authors have addressed the concerns raised by the previous reviewers. The contexts and arguments placed in the paper are convincing. Water availability to the requirements of specific crops is the prime production condition, absence of which, could nullify contributions of other inputs. There are specific thresholds of optimal water requirement for every crop, which the precipitation and/or irrigation provisioning need to ensure in case water availability deviates from optimality. Thus, there involves optimization of costs for water provisioning, and all requires precise estimates on the cost and benefits derived from land use. These aspects though are not explicit in the paper, one could relate the significance the paper posits. The context of the study is thus presented well - a timely and accurate assessment of the drought situation (water availability assessment to the requirements of crops cultivated) is of great significance in sustaining production. The authors are well aware of the developments in the field of GIS and applications, consider a host of factors including the topography and human activities as drivers of drought. There is high water demand in the study area, though causes of demand are indicated, could be explained further as – water availability declines because of variation of precipitation, and higher water use intensity is because of cropping pattern change driven by the market. and higher human consumption of water in new areas of consumption.

Data sources are well indicated and methodology is well explained in the paper to measure the TVDI. Geodetector model however requires detailed justification on how all the factors act as drivers. If the results show drought reduction and enhancement of drought across various seasons, the explanatory factors need detailed discussion. For instance, the main reason for the gradual increase of drought in summer is temperature and increased need of water for crops, as because it is the prime growing season of crops. Further, the study area has high population density, and the demand for crops would be market driven, and market as a factor to intensify input use to derive higher yield and even change cropping patterns for exotic crops, which may require more water. Detailed discussion on these lines along with crop data could have made the analysis comprehensive.

Drought could be linked to cropping seasons, assessing water requirements for crops cultivated, and drought could be aggravated/reduced by introduction/withdrawal of water intensive crops. Mapping of crops to the seasons could have arrived to derive better results from the exercises. There is scope to have simulation exercises on water requirement on three fronts - cropping pattern to the ecological condition of the study area; introduction of water intensive crops driven by market, and introduction of low water intensive crops driven/compensated by state subsidy; and as indicated technology can complement/aggravate the rest of the interventions to ensure optimality or aggravating the droughts too.

Answer: Thank you for your suggestions.

First, regarding your comment that "The Geodetic model needs to specify how all the factors act as drivers," in Section 2.3.4 of the paper, we added tables to explain the driving logic of the different drivers of the Geodetector model. Relevant references have also been added to make it easier for readers to read and understand. **Please refer to lines 235-238.**

Second, regarding the suggestions you mentioned about "changes in planting structure caused by market demand, increasing water consumption in irrigated areas" and "it is recommended to introduce low-water consumption crops according to national policies," we have adopted your suggestions when revising the discussion part of this article. Section 4.1 and Section 4.3 have been supplemented and modified to improve the comprehensiveness of the discussion and enhance the depth of the paper. **Please refer to lines 488-538, 565-582.**

**Reviewer #7**

The research study provides a detailed, well-structured analysis of drought trends in the Xinjiang Santun River Irrigation District, integrating remote sensing and statistical models. The study effectively identifies spatial heterogeneity, emphasizing that the northern part of the irrigation area experiences more severe drought than the southern part. However, some revisions and improvements are needed in the study before publication. By addressing these suggestions, the study will meaningfully contribute to drought risk assessment, water resource management, and climate change adaptation strategies in arid agricultural regions.

1. The study categorizes drought severity but does not explain the basis and threshold values for these classifications (e.g., what threshold defines “moderate” vs. “severe” drought?)

Answer: Thank you for your review. Regarding the question you mentioned, I would like to give you a detailed explanation. The establishment of the TVDI threshold system in this study is based on the following three bases.

1) **Theoretical basis:** According to the physical significance of the TVDI in the LST-NDVI feature space (Sandholt et al., 2002), when TVDI>0.6, the soil water supply is lower than the critical value of the vegetation water demand, indicating that drought stress begins to significantly affect the physiological process of the vegetation.

2) **Regional verification:** We determined the classification threshold in the paper by referring to the relevant research on arid areas and fitting the measured soil water content in the irrigation area. In order to prevent misunderstanding, we have added a table that presents the classification in the paper and have added an explanation of the drought degree.

3) **The calibration of historical events:** The research results were compared with the statistical yearbook of drought disasters in Changji, Xinjiang. More than 80% of the regions were found to match agricultural disaster reports of crop loss.

However, due to space limitations, we briefly describe the classification logic in this paper and note the references. In addition, descriptions of the phenomena of different drought levels have been added in Table 3. **Please refer to lines 182-187.** In order to prevent readers from misunderstanding, in the discussion section, we present an in depth discussion of the current limitations of the TVDI indicators and the uncertainties introduced by remote sensing data clouds and sensors.**Please refer to lines 584-603.**

2. There is a need to discuss the potential bias in TVDI estimates, especially in sparsely vegetated regions where NDVI approaches zero and may fail to represent soil moisture conditions.

Answer: According to your suggestion, a discussion of the limitations of the TVDI indicator has been added to subsection 4.4. **Please refer to lines 584-603.**

3. The study needs to discuss the model uncertainty and how the data limitations (like- cloud cover, sensor errors) may affect the results.

Answer: We entirely agree with you. We preprocessed all of the remote sensing images utilized in this study, including radiometric calibration, atmospheric correction, and cloud removal, to ensure that all of the data cloud sizes were less than 10%, and the data quality was high.

Based on your suggestion, discussion of the different factors affecting the accuracy of the model has been added to Section 4.4 of the paper. **Please refer to lines 584-586.**

4. Data on groundwater depletion needs to be incorporated to establish the claim of over-extraction.

Answer: According to your review, we have added references on groundwater over-extraction appropriate places in the paper. **Please refer to lines 518-519.**

5. The study needs to discuss how various irrigation policies and water allocation strategies implemented in the area affect drought severity.

Answer: We have reorganized the discussion section according to your suggestion. We have appropriately increased the discussion of the irrigation policies and unequal water distribution, which contribute to drought. **Please refer to lines 519-521.**

**Reviewer #8**

Author need to check and use concise language to enhance readability for readers. Also needs recheck the PLOS ONE Guidelines thats why requires some minor corrections before it can be accepted for publication.

Manuscript Title: “Analysis of growing season drought characteristics and driving factors for vegetation in the Santun River Irrigation Area in Xinjiang”.

Manuscript ID: PONE-D-24-49768R1

Dear Authors,

I enjoyed reading this work. However, I have just some minor comments which are given section-wise.

The manuscript addresses a very relevant matter on the growing season drought characteristics and driving factors for vegetation. It highlights some pertinent issues in the context of agricultural production and the economic crisis of the global south region. Therefore, The topic is interesting and could be an important contribution to the journal and the discipline of climatic paradigm and agricultural production. However, the manuscript requires some minor corrections before it can be accepted for publication.

General comments:

1. Use concise language to enhance readability for readers.

Answer: Thank you for your suggestions. We have revised the paper as a whole with reference to the comments of the reviewers and have enhanced the conciseness of the article. We have hired a professional language editing service to thoroughly check and touch up the manuscript.

2. Research gap needs to be justified with the help of previous literature.

Answer: According to your suggestions, we have rearranged the parts of the article that need to be referenced and have added corresponding citations to prove that the current research is novel and valuable. **Please refer to lines 34-86, 491, 496, 505, 509, 519, 525, 535, 536, 542, 549, 558, 561, and so on.**

3. The methods section should be written clearly and comprehensively to ensure accessibility and understanding for readers. Basically who are without a background of quantitative research.

Answer: Based on your suggestions, we have reorganized the methods section, adding appropriate reasons for why each method was chosen, as well as the advantages and principles of each method, to make it easier for readers to read and understand. **Please refer to lines 171-175, 189-193, 212-215, 221-225.**

4. Methodology portions needed to be written sequentially and needed to be justified with previous literature review.

Answer: Based on your suggestions, we re-examined the methodological sections and wrote them strictly in the order of the studies. The research sequence is as follows: drought index, trend analysis, spatial transfer analysis, and driving factor analysis. The methodology section is now written in strict accordance with this framework. In addition, we introduce the principle and reasons for the selection of each research method and cite relevant literature to demonstrate. **Please refer to lines 171-175, 189-193, 212-215, 221-225.**

5. More policy recommendations need to be discussed by underpinning the findings.

Answer: Based on your suggestion, we have rearranged the strategic suggestions in the discussion section based on the existing research results. **Please refer to lines 565-582.**

**Abstract:**

The abstract provides an overview of the research topic but lacks of conciseness – by following the structure sequentially like background, objectives, methods, results, and conclusions would provide a standard structure of the abstract.

Answer: Based on your suggestion, we have rewritten the summary in strict accordance with the framework of "background, purpose, methods, results, and conclusion" to make it easier for the readers to read and understand the content. **Please refer to lines 14-30.**

**Introduction:**

1. Overall, the introduction portion is comprehensive, but the sentences lack of proper linkages. Therefore, the suggestion is to critically evaluate the literature to highlight specific gaps by incorporating more recent studies to reflect advancements in the climatic paradigm, including human aspects.

Answer: Based on your suggestions and those of the other reviewers, we have revised the introduction and have included more recent research results. In addition, the specific research objectives of this paper are clearly pointed out. **Please refer to lines 34-109.**

**Study Area:**

1. After replacing this “Study Area, Data, and Methods” authors should write study area only in the line of 96.

Answer: This has been modified accordingly.**Please refer to line 110.**

2. The paragraph needs to include why Xinjiang Santun River Irrigation District is more important than others in the context of climatic chapters with a proxy of drought.

Answer: Based on your suggestion, we have revised the outline of the study area to emphasize that the study area is a typical arid land oasis agricultural system. **Please refer to lines 112-124.** In addition, in the introduction, we have also appropriately stated the importance of the study area. **Please refer to lines 87-91.**

Regarding the "Why the Santun River irrigation area of Xinjiang is more important than other irrigation areas?" the main reasons are discussed below.

First, the Santun River Irrigation area in Xinjiang is located in the arid zone on the north slope of the Tianshan Mountains where water resources are scarce and the ecosystem is fragile. Drought has a significant impact on agricultural production and the ecological environment in this area. In addition, we also pointed out in the discussion section of this paper that the land use types in this region have also undergone significant changes during the past 20 years, and this nonlinear interaction between natural and human factors provides an ideal case for analyzing the driving mechanism of drought.

Second, the Santun River Irrigation Area in Xinjiang, as the core area of the agricultural economy in Changji City, Xinjiang, is mainly dominated by high water consumption crops such as cotton and wheat. By studying the spatiotemporal differentiation of the drought in this area, the response threshold of an oasis agricultural system to climate change can be revealed, providing a scientific basis for optimizing the irrigation system and planting structure.

Finally, most existing studies focused on large-scale regions (such as the North China Plain and the Tibetan Plateau), while there are relatively few detailed studies on key eco-economic synergies such as the north slope of the Tianshan Mountains. Our research on the Santun River irrigation area fills the regional gap in drought-driver research and provides a reference for sustainable development in similar arid areas.

**Data and Methods:**

1. “In line 116 the title “Data sources and Processing” should be corrected as “Material and Methods”.

Answer: This has been modified accordingly. **Please refer to line 127.**

2. In line 130 the title “Methods” should be corrected as “Methods of Data Analysis”.

Answer: This has been modified accordingly. **Please refer to line 159.**

3. Minimal discussion has been done on how the data collection methods ensure reliability and validity. So, the suggestion is to discuss potential biases and how they are allayed in the data collection process by citing previous literature.

Answer: Based on your suggestions, we have appropriately added references related to the data collection methods and image preprocessing. **Please refer to lines 128-134.** In addition, we now discuss the possible deviations of the data collection in the discussion section.**Please refer to line 584-586.**

**Results and Discussion:**

1. To improve the quality of work, findings should be analyzed in reference to a theoretical framework. Include a comparison discussion with similar studies to contextualize the results in the same periphery.

Answer: According to your suggestions, we have reorganized the discussion section and conducted a block discussion according to the paper’s framework. In addition, in the discussion section, we introduce more similar studies for demonstration purposes. **Please refer to lines 488-582.**

**Conclusions:**

1. A concise summary should be included in the conclusion, along with key points from the results and discussion, while also addressing the study's limitations to provide a comprehensive closing perspective for this study.

Answer: Based on your suggestion, we have revised the conclusion to summarize the results of the entire study in more concise language. In addition, in the last paragraph of the conclusions section, we make appropriate reference to the limitations of policies and research on drought response. **Please refer to lines 620-643.**

**Responses to comments on the annex**

Overall, this research piece of work is very relevant in the context and time of climate change because it primarily impacted humans negatively. Still, there is a requirement for some change to make this piece of work more academically sound for the reader.

1. In the abstract, the significance and conclusion sections are given less emphasis, typically appearing at the end. Meanwhile, the results and methodology sections are overly detailed. To balance the abstract, some content from these sections should be condensed and incorporated into the significance and conclusion.

Answer: Thank you for your review. We have rewritten the abstract in strict accordance with the framework of "background, purpose, methods, results, and conclusions." In addition, we have appropriately reduced the introduction of the methods and balanced the structure of the abstract to make it easier for readers to read and understand. **Please refer to lines 14-30.**

2. In the Introduction, the discussion lacks literature on the driving forces of drought and its impact on agriculture and human development. Instead, it focuses only on transitioning to new methods/models. Given the title's emphasis on driving forces, incorporating this aspect would enhance clarity and provide a better understanding for readers.

Answer: Based on your suggestions, we have rewritten the introduction to appropriately add research on the driving forces of drought. The research goals of this study are now clearly pointed out, enabling readers to more easily read and understand. **Please refer to lines 65-75.**

3. A strong rationale is needed for using NDVI and TVDI in this study. Why are these indices essential, and how does this study contribute to your research area? While TVDI is widely used, its limitations in sparsely vegetated or bare soil regions should be explicitly acknowledged. Additionally, comparing it with other drought indices (SPI, SPEI, VHI) would enhance clarity.

Answer: Thank you for your review. We would like to provide detailed answers to the questions you raised.

Regarding the issue that "the use of TVDI in this article needs a clear reason and should be compared with indicators such as SPI and SPEI," we have rewritten the introduction section to clearly state that traditional drought indicators (such as the SPI, SPEI, and PDSI) all have drawbacks. In addition, compared with them, the TVDI has a more explicit physical mechanism for drought inversion through the LST-NDVI feature space, which can effectively eliminate the influence of single factors on the monitoring results. Moreover, the model construction is simple and the effect is significant. Therefore, it is more suitable for monitoring agricultural ecosystems. Thus, we chose the TVDI as the drought indicator to quantify the drought situation in the study area. To make it easier for readers to read and understanding the content, we have explained the reasons for choosing each research method and have provided corresponding references as evidence. **Please refer to lines 44-53, 171-175.**

Regarding the question of "What contributions does this research make to my research field?", this paper mainly takes the San Tun River Irrigation District in Xinjiang as an example, and by means of a combination of trend analysis and the geographical detector model, the spatiotemporal evolution characteristics and driving mechanisms of drought in the study area are analyzed. The research results provide a theoretical basis for drought risk assessment and water resource scheduling in arid regions, and also provide a reference for drought monitoring research in similar areas. In addition, the San Tun River Irrigation District in Xinjiang is a typical irrigation district in an arid area with scarce water resources and a fragile ecosystem. Moreover, the discussion section of this paper also points out that the land use types in this area have undergone significant changes during the past 20 years. The nonlinear interactions between the natural and human factors also provide an ideal case for analyzing the driving mechanisms of drought.

Regarding the issue of "defects of TVDI indicators," to prevent readers from misunderstanding, we present an in-depth analysis and discussion of this issue in the discussion part of the paper. **Please refer to lines 44-53, 586-603.**

4. The interaction between elevation and temperature (q-value = 0.869) is identified as the strongest factor, but the underlying physical mechanisms are not well explained also the Geo Detector results lack significance testing (p-values, confidence intervals), which should be included for better validation.

Answer: Thank you for your review. After discussion among the team, the physical mechanism behind the interaction between the DEM and temperature has been reorganized and rewritten, and relevant references have been added to demonstrate the points made in the paper. **Please refer to lines 551-563.**

Regarding the problem of "lack of significance test of the results of the geographical detector," I present a detailed answer here. In the analysis of two-factor interactions, the geographical detector model does not provide a P-value test. The main reasons are discussed below.

1) Core functions of the geographic detector: The main objective of the geographic detector model is to quantify the spatial differentiation explanatory power of factors on dependent variables through q-values and to evaluate the synergistic influence of multiple factors through interaction detection. The interaction analysis mainly focuses on the change in the q-value, rather than the traditional statistical significance test.
2) Interaction type judgment: In interaction detection, by comparing the single-factor q-value (q(X1), q(X2)) with the two-factor interaction q-value (q(X1∩X2)), the type of interaction (such as enhanced, diminished, and independent) between the factors can be judged. This analysis focuses more on the direction and strength of interactions than on their significance.
3) Limitations of significance testing: In interaction analysis, significance testing is not the core objective. The complexity of interactions (such as nonlinear relationships) can make traditional significance tests difficult to apply, and therefore, a common two-factor interaction analysis will not provide a p-value.

In summary, the p-value test is not listed in the two-factor interaction analysis in this study. However, in order to demonstrate the reliability of the results, the significance test results have been added to the single factor detection analysis in this study, and are now presented in Table 7 in the results analysis. To better support the above answer, I have listed some references below.

[1] Guo Q, Lai X, Jia Y, et al.Spatiotemporal Pattern and Driving Factors of Carbon Emissions in Guangxi Based on Geographic Detectors[J].Sustainability (2071-1050), 2023, 15(21).DOI:10.3390/su152115477.

[2] Wang M, Chen Y, Li J, et al. Spatiotemporal evolution and driving force analysis of drought characteristics in the Yellow River Basin [J]. Ecological Indicators, 2025, 170 113007-113007.DOI: 10.1016/j.ecolind.2024.113007

[3] Wang X, Meng Q, Zhang L, et al.Evaluation of urban green space in terms of thermal environmental benefits using geographical detector analysis[J].International Journal of Applied Earth Observation and Geoinformation, 2021.DOI:10.1016/j.jag.2021.102610.

5. Why did you select only the 2005–2023 period? Provide a clear justification for this timeframe, explaining its relevance to the study. Clarifying this choice will also strengthen the trend analysis.

Answer: Regarding the question "Why study period: 2005–2023?", the three main reasons are discussed below.

1) Data integrity and continuity: The Landsat series of satellites has been providing remote sensing data with global coverage since 1972. The Landsat 5, 7 and 8 satellites have been in orbit since 2005, and these Landsat data provide high-quality, long-term image data to ensure research continuity and data integrity. In the data preparation stage of this study, we found that before 2005, some of the data from the Landsat series satellites are missing, which made it difficult to ensure the reliability and integrity of the research results. The paper was written in May 2024, and the use of the data for 2023, which are the latest data for integer years, ensures that the research conclusions reflect the latest drought situation in the irrigation area.
2) Background of climate change: Since 2005, drought events have occurred frequently and have had significant impacts in Xinjiang. The choice of this time period allows the results to better reflect the influence of climate change on the temporal and spatial characteristics of drought evolution in the Santun River irrigation District, Xinjiang.
3) Background of the study area: Xinjiang is an important agricultural production base in China, and the frequent droughts in this area have had huge impacts on the regional ecology and social economy. During 2005–2023, Xinjiang took a series of measures related to water resource management and ecological protection. Studying the characteristics and influencing factors of drought during this period is of great significance for evaluating the effects of these measures and optimizing future water resource management strategies.

In summary, the choice of 2005–2023 as the research period not only meets the needs of data integrity and continuity but also better reflects the impacts of climate change and human activities on drought in recent years and provides a scientific basis for water resource management and ecological protection in arid irrigated areas. In order to prevent readers from having doubts, we re-emphasize the importance of the study area in the introduction, and we explain that since 2005, a series of measures related to water resource management and ecological protection have been taken in the irrigation area. **Please refer to lines 87-99.**

6. The study links severe drought in the northern region to natural factors but overlooks human-induced impacts like groundwater extraction and irrigation inefficiencies. It should also discuss local drought mitigation efforts. Additionally, the conclusion needs revision to restate the topic and provide concrete recommendations

Answer: According to your suggestion, we have reorganized the discussion section of the paper, and discussion of the influence of human factors on drought has been added to Section 4.1. In addition, we added a description of the causes of groundwater overextraction and low irrigation efficiency, and we also introduce relevant references to further support the discussion. **Please refer to lines 488-538.**

According to your suggestions, the conclusions of this article have been revised to delete unnecessary expressions, condense the language expression, and appropriately add specific suggestions in the last paragraph. **Please refer to lines 620-643.**

**Recommendation**

Minor Revisions Required: The study is valuable but would benefit from clearer justification of TVDI, stronger discussion of drought-driving mechanisms, and better statistical validation of GeoDetector results. Addressing these points will significantly enhance the manuscript’s clarity and impact.

Answer: Thank you for your review. We have revised the article in detail according to the comments of the reviewers, and we have hired a professional language editing service to thoroughly check and polish the manuscript.

Regarding your comment on the "reasons for increasing the use of TVDI," we have added specific reasons for using each method in the methodology section of this paper and have added references to relevant literature for support.**Please refer to lines 171-175.**

Regarding your comment on "adding discussion on the driving mechanism behind drought," we have made detailed revisions according to the comments of the reviewers, and we now discuss the driving mechanism behind drought in more depth. In addition, corresponding references have been added to support the relevant discussion. **Please refer to lines 551-563.**

In terms of "adding statistical verification of the results of GeoDetector," we have introduced p-value tests in the single-factor detection analysis. The reasons for not adding p-value tests in the two-factor interaction analysis are discussed below.

1) Core functions of geographic detector: The main objective of the geographic detector model is to quantify the spatial differentiation explanatory power of factors on dependent variables through q-values and to evaluate the synergistic influence of multiple factors through interaction detection. The interaction analysis mainly focuses on the change in the q-value, rather than the traditional statistical significance test.
2) Interaction type judgment: In interaction detection, by comparing the single-factor q-value (q(X1), q(X2)) with the two-factor interaction q-value (q(X1∩X2)), the type of interaction (such as enhanced, diminished, and independent) between he factors can be judged. This analysis focuses more on the direction and strength of interactions than on their significance.
3) Limitations of significance testing: In interaction analysis, significance testing is not the core objective. The complexity of interactions (such as nonlinear relationships) can make traditional significance tests difficult to apply, and therefore, a common two-factor interaction analysis will not provide a p-value.

In summary, the p-value test is not listed in the two-factor interaction analysis in this study. However, to demonstrate the reliability of the results, we added the significance test results to the single-factor detection analysis in this study and are marked in Table 7 in the results analysis part. Moreover, to better support the above answer, we have listed some references below.

[1] Guo Q, Lai X, Jia Y, et al.Spatiotemporal Pattern and Driving Factors of Carbon Emissions in Guangxi Based on Geographic Detectors[J].Sustainability (2071-1050), 2023, 15(21).DOI:10.3390/su152115477.

[2] Wang M, Chen Y, Li J, et al. Spatiotemporal evolution and driving force analysis of drought characteristics in the Yellow River Basin [J]. Ecological Indicators, 2025, 170 113007-113007.DOI: 10.1016/j.ecolind.2024.113007

[3] Wang X, Meng Q, Zhang L, et al.Evaluation of urban green space in terms of thermal environmental benefits using geographical detector analysis[J].International Journal of Applied Earth Observation and Geoinformation, 2021.DOI:10.1016/j.jag.2021.102610.
